# Implication of granulocyte-macrophage colony-stimulating factor induced neutrophil gelatinase-associated lipocalin in pathogenesis of rheumatoid arthritis revealed by proteome analysis

**DOI:** 10.1186/ar2587

**Published:** 2009-01-08

**Authors:** Masayoshi Katano, Kazuki Okamoto, Mitsumi Arito, Yuki Kawakami, Manae S Kurokawa, Naoya Suematsu, Sonoko Shimada, Hiroshi Nakamura, Yang Xiang, Kayo Masuko, Kusuki Nishioka, Kazuo Yudoh, Tomohiro Kato

**Affiliations:** 1 Institute of Medical Science, St Marianna University School of Medicine, 2-16-1 Sugao, Miyamae-ku, Kawasaki, Kanagawa 216-0015, Japan; 2 Department of Advanced Medicine Development, Mitsubishi Chemical Medience Corporation, 4-2-8 Shibaura, Minato-ku, Tokyo 108-8559, Japan; 3 Clinical Proteomics & Molecular Medicine, St Marianna University Graduate School of Medicine, 2-16-1 Sugao, Miyamae-ku, Kawasaki, Kanagawa 216-0015, Japan; 4 Department of Joint Disease and Rheumatism, Nippon Medical School, 1-1-5 Sendagi, Bunkyo-ku, Tokyo 113-8602, Japan; 5 Department of Rheumatology and Immunology, University Hospital, Hubei University for Nationalities, 11 Xueyuan Road, Wuchang, Wuhan, Hubei 430062, PR China

## Abstract

**Introduction:**

In rheumatoid arthritis (RA), synovial fluid (SF) contains a large number of neutrophils that contribute to the inflammation and destruction of the joints. The SF also contains granulocyte-macrophage colony-stimulating factor (GM-CSF), which sustains viability of neutrophils and activates their functions. Using proteomic surveillance, we here tried to elucidate the effects of GM-CSF on neutrophils.

**Methods:**

Neutrophils stimulated by GM-CSF were divided into four subcellular fractions: cytosol, membrane/organelle, nuclei, and cytoskeleton. Then, proteins were extracted from each fraction and digested by trypsin. The produced peptides were detected using matrix-assisted laser desorption ionisation-time-of-flight mass spectrometry (MALDI-TOF MS).

**Results:**

We detected 33 peptide peaks whose expression was upregulated by more than 2.5-fold in GM-CSF stimulated neutrophils and identified 11 proteins out of the 33 peptides using MALDI-TOF/TOF MS analysis and protein database searches. One of the identified proteins was neutrophil gelatinase-associated lipocalin (NGAL). We confirmed that the level of NGAL in SF was significantly higher in patients with RA than in those with osteoarthritis. We next addressed possible roles of the increased NGAL in RA. We analysed proteome alteration of synoviocytes from patients with RA by treatment with NGAL *in vitro*. We found that, out of the detected protein spots (approximately 3,600 protein spots), the intensity of 21 protein spots increased by more than 1.5-fold and the intensity of 10 protein spots decreased by less than 1 to 1.5-fold as a result of the NGAL treatment. Among the 21 increased protein spots, we identified 9 proteins including transitional endoplasmic reticulum ATPase (TERA), cathepsin D, and transglutaminase 2 (TG2), which increased to 4.8-fold, 1.5-fold and 1.6-fold, respectively. Two-dimensional electrophoresis followed by western blot analysis confirmed the upregulation of TERA by the NGAL treatment and, moreover, the western blot analysis showed that the NGAL treatment changed the protein spots caused by post-translational modification of TERA. Furthermore, NGAL cancelled out the proliferative effects of fibroblast growth factor (FGF)-2 and epidermal growth factor (EGF) on chondrocytes from a patient with RA and proliferative effect of FGF-2 on chondrosarcoma cells.

**Conclusions:**

Our results indicate that GM-CSF contributes to the pathogenesis of RA through upregulation of NGAL in neutrophils, followed by induction of TERA, cathepsin D and TG2 in synoviocytes. NGAL and the upregulated enzymes may therefore play an important role in RA.

## Introduction

Rheumatoid arthritis (RA) is a chronic inflammatory polyarthritis, characterised by a proliferation of synovial cells and infiltration of inflammatory cells into the synovium. In RA, synovial fluid (SF) contains a large number of neutrophils, which are attracted from the synovial microstructure to the synovial cavity by chemotactic agents such as C5a and leukotriene B [1]. The neutrophils in SF make contact with immune complexes and digest them by phagocytosis. This process activates neutrophils. The activated neutrophils are characterised by a high level expression of CD69, since CD69 is located intracellulary in neutrophils at a resting state and moves rapidly to the cell surface upon stimulation with phorbol myristate acetate or *N*-formylmethionine leucyl-phenylalanine [2]. The activated neutrophils release reactive oxygen species [3,4], cytokines such as interleukin (IL)1 and IL8 [5] and proteases [6], leading to the inflammation and destruction of the joints in RA.

Development of neutrophils from haematopoietic stem cells involves several cytokines. In particular, granulocyte colony-stimulating factor (G-CSF) maintains neutrophil production at steady state and increases production of neutrophils in emergency situations [7,8]. By contrast, granulocyte-macrophage colony-stimulating factor (GM-CSF) sustains the viability of neutrophils and activates their functions. For example, GM-CSF primes neutrophils via phosphorylation of p47phox for the activation of nicotinamide adenine dinucleotide phosphate (NADPH) oxidase, which produces superoxide anions [9]. Further, GM-CSF increases the activity of extracellular signal-regulated kinase (ERK) and delays apoptosis, possibly through the activation of Lyn kinase [10,11]. In addition, GM-CSF stimulates neutrophils to express CD69 activation marker on their surface [12]. Clinically, GM-CSF has been reported to be produced at high levels from synoviocytes of patients with RA *in vitro *[13] and, in fact, GM-CSF has been detected in SF from patients with RA [14]. Thus, GM-CSF possibly contributes to inflammation and destruction of joints in RA through neutrophil activation. Therefore, it would be of great help in understanding the pathogenesis of RA to clarify the effects of GM-CSF on neutrophils. In the present work, we have tried to elucidate the novel effects of GM-CSF on neutrophils by using proteomic surveillance.

Proteomic surveillance methods, which have recently showed prominent advances, are roughly divided into two types. The first is direct use of mass spectrometry, and the other is the combination of two-dimensional electrophoresis (2-DE) and mass spectrometry (MS). Here, we first used matrix-assisted laser desorption ionisation-time-of-flight (MALDI-TOF) MS to detect neutrophil peptides upregulated by GM-CSF. This technique is reliable, as we recently used it to successfully detect disease-specific short peptides in systemic sclerosis [15]. We next used 2DE-MS to elucidate effects of one of the GM-CSF-affected proteins, neutrophil gelatinase-associated lipocalin (NGAL), on synoviocytes. NGAL has been reported to be stimulated by GM-CSF in [^35^S]methionine metabolic studies [16]. Our present proteomic surveillance study confirmed the upregulation of NGAL by GM-CSF in neutrophils. Further, our present study found that stimulation of RA synoviocytes by NGAL enhanced production of transitional endoplasmic reticulum ATPase (TERA), cathepsin D, and transglutaminase 2 (TG2). Additionally, NGAL abolished the proliferative effects of fibroblast growth factor (FGF)-2 and epidermal growth factor (EGF) on chondrocytes from a patient with RA, and the proliferative effect of FGF-2 on chondrosarcoma cells.

## Materials and methods

### Cells and clinical samples

Human neutrophils were separated by dextran sedimentation and Ficoll-Hypaque (GE Healthcare Bioscience, Piscataway, NJ, USA) density-gradient centrifugation [17] from peripheral blood of healthy volunteers. A chondrosarcoma cell line of OUMS-27 [18] was obtained from Health Science Research Resources Bank of Japan (Cell number, IFO50488).

Synoviocytes were prepared from synovial tissue samples obtained from 62-year-old and 73-year-old women with RA, and chondrocytes were obtained from a 72-year-old woman with RA during knee joint arthroplasty. Synovial fluid samples were obtained from 13 patients with RA (13 women, 0 men; aged 59 to 84 years old, mean age 70.7 years) and 13 patients with osteoarthritis (OA) (10 women, 3 men; aged 55 to 89 years old, mean age 69.0 years). The patients were diagnosed according to the respective classification criteria for each of the two diseases [19,20]. All the clinical samples were obtained after the patients gave their informed consent, and this study was approved by the local institutional ethics committee.

### Stimulation of neutrophils with GM-CSF and proteome analysis by MALDI-TOF MS

The purified neutrophils were resuspended in RPMI 1640 containing 10% foetal bovine serum (FBS), 100 U/ml penicillin, 100 μg/ml streptomycin, and 2 mM glutamine. The neutrophils were cultured in the presence or absence of 400 U of recombinant human GM-CSF (Millipore, Billerica, MA, USA)/10^7^cells under 5% CO_2 _at 37°C for 18 h [12]. Then, the neutrophils were divided into four fractions: cytosol, membrane/organelle, nuclei and cytoskeleton, and proteins were extracted from each of the fractions using Subcellular Proteome Extraction Kit (Merck, Rahway, NJ, USA), according to the manufacturer's instructions. Each of the four protein fractions was digested by trypsin (Promega, Madison, WI, USA) for 3 h. The trypsin-digested peptides, concentrated by Ziptip C18 (Millipore), were placed on the anchor chip of a MALDI-TOF mass spectrometer (Ultrafrex, Bruker Daltonics, Bremen, Germany) together with 100 fmol of a bradykinin fragment (m/z of 757) (Sigma, St Louis, MO, USA) as an internal control and 0.3 μg of 4-hydroxy-α-cinnamic acid matrix.

Next, mass spectra of peptide peaks were detected using the automatic linear positive mode for simple comparison between the sample groups. The MS analysis was then performed using reflector mode to obtain accurate masses for the peptides. Finally, the MS/MS (TOF/TOF) analysis and subsequent sequence searching using Mascot [21] were performed to identify the sequences of peptides of interest. A comparative analysis of the mass spectra of the peptide peaks between the GM-CSF-treated and the untreated samples was performed by using ClinProt Tools software v. 1.0 (Bruker Daltonics) as previously described [15]. The intensities of the detected peptides were normalised using that of the bradykinin fragment.

### Western blotting

The cultured neutrophils were solubilised in lysis buffer containing 30 mM Tris-HCl, pH 8.5, 4% 3-((3-cholamidopropyl)dimethylammonio)propanesulfonate (CHAPS), 7 M urea, and 2 M thiourea. After centrifugation for 30 min at 14,000 *g*, the supernatant was used for separation by 12.5% sodium dodecyl sulfate (SDS)-polyacrylamide gel electrophoresis (PAGE). The separated proteins were then transferred to nitrocellulose membranes. After blocking for 1 h in phosphate buffered saline (PBS) containing 1% bovine serum albumin and 0.1% Tween-20, the membrane was incubated for 1 h with a rat monoclonal anti-human NGAL antibody (R&D Systems, Minneapolis, MN, USA), followed by incubation with horseradish peroxidase (HRP)-conjugated goat anti-rat IgG antibodies. Immunoreactive bands were detected by using 3,3'-diaminobenzidene (DAB) and H_2_O_2_.

### Real-time polymerase chain reaction (PCR)

Total RNA was isolated from the cultured neutrophils using an RNeasy mini kit (Qiagen, Hilden, Germany). Reverse transcription of mRNA was performed using oligo-dT primers (Invitrogen, Carlsbad, CA, USA) and SuperScript II reverse transcriptase (Invitrogen). The produced cDNA were used as templates for quantitative PCR amplification. The sequences of the primers used were as follows: NGAL (forward) 5'-gtaggcctggcagggaatg-3'; NGAL (reverse) 5'-ggaacaaaagtcctgatccagtagtc-3'; glyceraldehyde 3-phosphate dehydrogenase (GAPDH) (forward) 5'-aatggaaatcccatcaccatctt-3'; GAPDH (reverse) 5'-catcgccccacttgattttg-3'. PCR was performed using a LightCycler FastStart DNA Master SYBR Green I (Roche Diagnosis, Mannheim, Germany). The expression of mRNA for NGAL was normalised by that of a constitutively expressed housekeeping gene of GAPDH, and the values are expressed as a ratio of NGAL/GAPDH.

### Quantitation of NGAL in synovial fluids by ELISA

Concentrations of NGAL in the synovial fluid of patients with RA and of patients with OA were measured using a commercially available ELISA kit (Antibodyshop, Gentofte, Denmark) according to the manufacturer's instructions.

### Preparation of total protein from the cultured synoviocytes and protein labelling

The separated synoviocytes were cultured in Ham's nutrient mixture F-12 containing 10% FBS, 100 U/ml penicillin, and 100 μg/ml streptomycin. After two passages, almost all the cells were fibroblast-like synoviocytes (type A synoviocytes), as judged by microscopic observations. The cells were treated with or without 10 μg/ml of recombinant human NGAL (R&D Systems) under 5% CO_2 _at 37°C for 48 h. After two washes in PBS, the cells were dissolved in a lysis buffer containing 30 mM Tris-HCl (pH 8.0), 4% CHAPS, 7 M urea, and 2 M thiourea for 2-DE analysis. The extracted proteins were labelled with saturation dyes of Cy3 and Cy5 according to the manufacturer's instructions.

### Two-dimensional differential gel electrophoresis (2D-DIGE) analysis and protein identification

The labelled proteins were separated by 2D-DIGE as described previously [22]. Briefly, 2.5 μg of each protein sample of synoviocytes treated or untreated by NGAL was reduced with 2 nmol of Tris (2-carboxyethyl)-phosphine hydrochloride (Molecular Probes, Eugene, OR, USA) for 1 h at 37°C. Subsequently, 4 nmol of Cy5 saturation dye, freshly dissolved in anhydrous *N*, *N*-dimethylformamide, was added and the reaction was incubated at 37°C for 30 min. The labelling reaction was terminated by addition of an equal volume of lysis buffer (7 M urea, 2 M thiourea, 4% CHAPS, 130 mM dithiothreitol (DTT), and 2.0% Pharmalyte pH 4–7 (GE Healthcare)). All the labelling procedures were carried out in the dark. For the internal standard, equal aliquots (2.5 μg) of each sample, untreated or treated with NGAL, were pooled and labelled with Cy3 saturation dye. Then, the saturation Cy3-labelled internal standard sample and each of the individual saturation Cy5-labelled proteins were mixed and diluted to a final volume of 450 μl. the labelled proteins were mixed and loaded onto a 24 cm Immobiline Dry-Strip covering the range of pH 4 to pH 7 (GE Healthcare) for isoelectric focusing (IEF) using IPGphor (GE Healthcare). After IEF, the strips were equilibrated in the equilibration solution (50 mM Tris-HCl, pH 8.8, 6 M urea, 30% glycerol, 2% SDS, 10 mg/ml DTT) for 15 min at room temperature. The equilibrated strips were placed on top of 12.5% SDS-PAGE slab gels and sealed with a solution of 0.5% (w/v) agarose. Separation of the proteins by 2-DE was performed using 12.5% SDS-PAGE. The separated labelled proteins were scanned at 100-μm resolution using an image analyser (Typhoon 9400 Imager, GE Healthcare) according to the manufacturer's instructions. The acquired gel images were analysed using Progenesis software (Perkin Elmer, Wellesley, MA, USA).

For identification of proteins, 2-DE gel fragments with approximately 1 mm in diameter, which corresponded to protein spots of interest by the image analysis, were recovered and washed in double-distilled water for 15 min. Then, the gel fragments were cut into small pieces and decoloured in 200 μl decolouring solution (25 mM ammonium hydrogen carbonate, 50% acetonitrile) at room temperature for 10 min. The gel pieces were rehydrated in 10 μl trypsin solution (50 mM ammonium hydrogen carbonate, 5 mM calcium chloride, 0.02 μg/μl trypsin) and incubated at 37°C for 16 h for digestion of the contained proteins with the trypsin. The digested peptides were extracted from the gel pieces using trifluoroacetic acid (TFA) and acetonitrile. Specifically, the digested products were supplemented with 50 μl of 5% TFA in 50% acetonitrile solution and vortexed. After centrifugation, the supernatant was recovered. After three more cycles of this extraction, the supernatant was filtered and concentrated down to 10 μl in an evaporator. The peptide sample solution was stored at -20°C until mass spectrometric analysis. Masses of the digested peptides in the samples were determined using a MALDI-TOF/TOF MS (Ultraflex, Bruker Daltonics). A list of the peptide masses determined was compiled for searching of the National Center for Biotechnology Information (NCBI) protein database using the Mascot software program (Matrix Science, London, UK).

### 2-DE separation and western blotting analysis of TERA

Synoviocytes were prepared from synovial tissue sample obtained from a 62-year-old woman with RA, and cultured as described above. After two passages, the cells were treated with or without 10 μg/ml of recombinant human NGAL for 48 h. Proteins were extracted and 100 μg of each protein sample from synoviocytes, treated or untreated with NGAL, were separated by 2-DE. The separated proteins were blotted onto a polyvinylidene difluoride membrane and detected with anti-TERA antibody (Affinity BioReagents, Golden, CO, USA) using ECL Advance western blotting detection reagents (GE Healthcare).

### Dimethylthiazol diphenyltetrazolium bromide (MTT) assay

OUMS-27, a human chondrosarcoma cell line, was cultured in DMEM containing 10% FBS, 100 U/ml penicillin, 100 μg/ml streptomycin, and 4 mM glutamine under 5% CO_2 _at 37°C. A total of 3 × 10^3 ^cells were seeded into each well of the 96-well plates. Then, the cells were treated with FGF-2 (1 ng/ml), and/or NGAL (1 μg/ml). After 0, 24, 48, and 96 h, the medium was replaced by a new batch containing MTT (0.5 mg/ml) and the cells were further incubated at 37°C for 4 h. Finally, the medium containing MTT was removed and 0.2 ml of 100% dimethylsulfoxide was added to each well. The absorbance was measured at 570 nm and at 650 nm as background subtraction.

Chondrocytes were prepared from cartilage tissue sample obtained from a 72-year-old woman with RA during knee joint arthroplasty, and cultured as described above. A total of 3 × 10^3 ^cells were seeded into each well of the 96-well plates. Then, the cells were treated with FGF-2 (1 ng/ml) or EGF (1 ng/ml), and/or NGAL (1 μg/ml) for 0, 48, and 72 h, and subjected to the MTT assay.

### Statistical analysis

Statistical significance was calculated by using the Student t test. A value of p < 0.05 was considered to be statistically significant.

## Results

### Proteome analysis of GM-CSF-treated or -untreated neutrophils by MALDI-TOF MS

**Figure 1:**
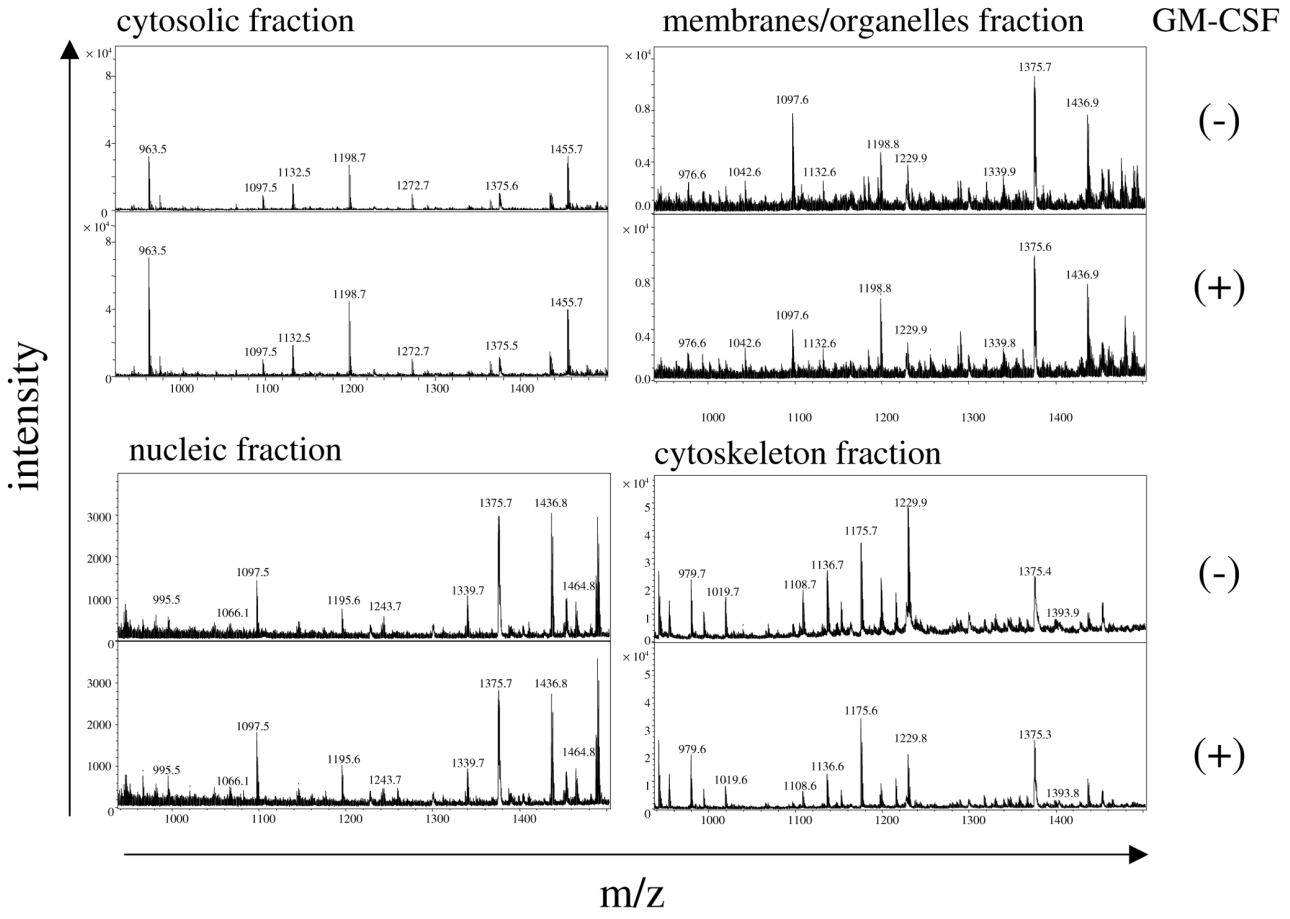
**Detection of trypsin digested peptides from granulocyte-macrophage colony-stimulating factor (GM-CSF) stimulated neutrophils by matrix-assisted laser desorption/ionization-time of flight mass spectrometry (MALDI-TOF MS)**. Neutrophils, treated with GM-CSF or untreated, were divided into four subcellular fractions: cytosol, membrane/organelle, nuclei and cytoskeleton. Then, proteins were extracted from each fraction and digested by trypsin. The produced peptides, concentrated by Ziptip C18, were placed together with a bradykinin peptide (m/z of 757) as an internal control on a chip of the MALDI-TOF MS. Representative spectra from 900 to 1,500 m/z are shown in each of the four fractions.

To understand effects of GM-CSF on resting neutrophils, we treated neutrophils obtained from healthy donors with GM-CSF for 18 h, at which point maximal CD69 induction by GM-CSF was observed [12], and then compared their proteome profile to that of untreated neutrophils using MALDI-TOF MS. First, we confirmed that GM-CSF induced activation of neutrophils by detecting CD69 on the cell surface using flow cytometry. On untreated neutrophils CD69 was undetectable, however the GM-CSF-treated neutrophils expressed CD69 strongly (data not shown). Next, we tried to ascertain whether GM-CSF affected neutrophil proteins by MALDI-TOF MS. We extracted total proteins from the GM-CSF-treated neutrophils and from the untreated neutrophils, digested them with trypsin and subjected the peptides produced to MALDI-TOF MS. Although many peptide peaks were detected, the intensities of the peaks were low (data not shown). Therefore, the differences between the peaks from treated and untreated neutrophils were poorly reproducible and the identification of the peptides by MS/MS analysis was confusing. Consequently, we divided the neutrophils into four subcellular fractions: cytosol, membrane/organelle, nuclei and cytoskeleton. Proteins extracted from each fraction were digested by trypsin, and the peptides produced subjected to MALDI-TOF MS. In this way, we successfully obtained representative peptide peak profiles as shown in Figure 1. We detected a total of 544 peptide spectra in the fractions. The intensities of the peptide peaks were normalised by the intensity of the bradykinin peptide fragment added as an internal control. Then, peptide peaks whose intensities were not less than 2.5-fold higher, or not more than 1 to 2.5-fold lower in GM-CSF-treated neutrophils than in untreated neutrophils, were selected for analysis. Using this method 47 peptide peaks (increase: 33, decrease: 14) were selected, as shown in Table 1.

**Table 1:** Peptide peak intensities increased or decreased by the treatment of granulocyte-macrophage colony-stimulating factor (GM-CSF)

m/z	Ratio (treated/untreated)	Fraction
2,176.0	5.0	Cytosol
2,216.0	3.3	Cytosol
763.4	3.1	Cytosol
2,042.1	2.9	Cytosol
963.5	2.9	Cytosol
2,726.4	2.8	Cytosol
2,008.9	2.8	Cytosol
2,138.2	2.7	Cytosol
1,882.9	2.7	Cytosol
854.4	2.7	Cytosol
795.5	2.7	Cytosol
1,515.7	2.6	Cytosol
1,883.9	2.6	Cytosol
1,630.8	2.5	Cytosol
825.2	7.8	Organelle/membrane
1,791.0	4.0	Organelle/membrane
841.2	3.7	Organelle/membrane
2,191.3	3.7	Organelle/membrane
845.2	3.0	Organelle/membrane
2,690.6	2.9	Organelle/membrane
861.2	2.8	Organelle/membrane
2,045.2	2.7	Organelle/membrane
1,954.2	2.7	Organelle/membrane
1,813.0	2.6	Organelle/membrane
711.4	2.6	Organelle/membrane
1,479.9	2.5	Organelle/membrane
2,384.2	2.5	Organelle/membrane
841.1	2.6	Nuclei
792.5	0.3	Nuclei
1,577.9	2.9	Cytoskeleton
1,562.0	2.8	Cytoskeleton
1,569.9	2.6	Cytoskeleton
1,584.0	2.6	Cytoskeleton
1,231.8	2.6	Cytoskeleton
1,810.0	0.4	Cytoskeleton
2,064.1	0.4	Cytoskeleton
743.1	0.4	Cytoskeleton
2,053.1	0.4	Cytoskeleton
2,036.1	0.4	Cytoskeleton
1,750.0	0.3	Cytoskeleton
1,536.0	0.3	Cytoskeleton
2,621.4	0.3	Cytoskeleton
1,772.3	0.3	Cytoskeleton
1,762.0	0.3	Cytoskeleton
2,152.2	0.3	Cytoskeleton
2,035.1	0.3	Cytoskeleton
2,015.2	0.3	Cytoskeleton

We then tried to identify these peptides by *de novo *sequencing using MS/MS analysis and subsequent protein database searching. We successfully identified amino acid sequences and parent proteins for 11 of the 47 peptide peaks, as shown in Table 2.

**Table 2:** Identification of the increased neutrophil proteins by the treatment of granulocyte-macrophage colony-stimulating factor (GM-CSF)

m/z	Protein	Ratio (treated/untreated)	Fraction	Accession no. (Swiss-Prot)
2,176.0	S100 calcium binding protein A9	5.0	Cytosol	[Swiss-Prot:P06702]
763.4	Neuropeptide S	3.1	Cytosol	[Swiss-Prot:P0C0P6]
963.5	S100 calcium binding protein A8	2.9	Cytosol	[Swiss-Prot:P05109]
854.4	NADH dehydrogenase 1 α subcomplex subunit 3	2.7	Cytosol	[Swiss-Prot:O95167]
795.5	Membrane-associated guanylate kinase	2.7	Cytosol	[Swiss-Prot:Q96QZ7]
1,515.7	Actin, β	2.6	Cytosol	[Swiss-Prot:P60709]
825.2	Ubiquitin-conjugating enzyme E2 E1	7.8	Organelle/membrane	[Swiss-Prot:P51965]
1,791.0	Neutrophil gelatinase-associated lipocalin (NGAL)	4.0	Organelle/membrane	[Swiss-Prot:P80188]
841.2	BMP-binding endothelial regulator protein	3.7	Organelle/membrane	[Swiss-Prot:Q8N8U9]
845.2	Glycoprotein M6-b	3.0	Organelle/membrane	[Swiss-Prot:Q13491]
1,480.0	FYVE, RhoGEF and PH domain-containing protein 4	2.5	Organelle/membrane	[Swiss-Prot:Q96M96]

BMP, bone morphogenetic protein; FYVE, phenylalanine (F)/tyrosine (Y)/valine (V)/glutamic acid (E) domain; Rho, Ras homolog; GEF, guanine nucleotide exchange factor; PH, pleckstrin homology.

### Confirmation of increased expression of NGAL in GM-CSF-treated neutrophils

Among the 11 identified proteins (Table 2), we focused on NGAL as it has been reported to be involved in the allosteric activation of matrix metalloproteinase (MMP)-9 [23-25], and in the protection of MMP-9 against degradation [23-25]. In fact, elevated serum levels of MMP-9 in RA have been reported [26].

**Figure 2:**
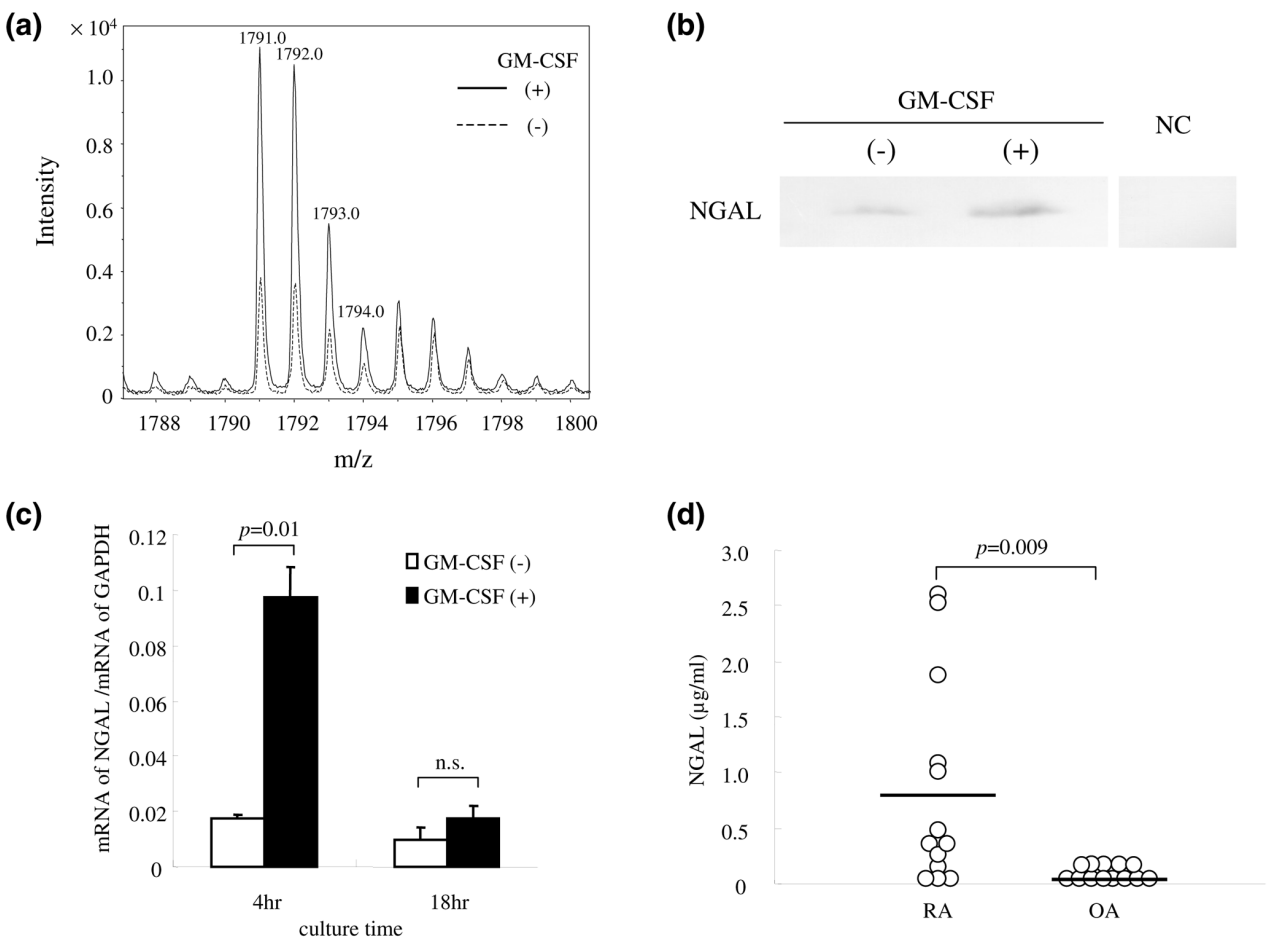
**Confirmation of increase of neutrophil gelatinase-associated lipocalin (NGAL) in neutrophils stimulated with granulocyte-macrophage colony-stimulating factor (GM-CSF) and in the synovial fluid of patients with rheumatoid arthritis (RA)**. **(a) **The intensity of the peptide with m/z 1,791.0, detected by matrix-assisted laser desorption/ionization-time of flight mass spectrometry (MALDI-TOF MS) and identified as NGAL by *de novo *sequencing using MS/MS and protein database searching, was compared between the organelle/membrane fractions of GM-CSF-treated and untreated neutrophils. **(b) **The increase of NGAL indicated by the mass spectrometric detection was further confirmed by western blotting using neutrophil lysate. Neutrophils treated with GM-CSF for 18 h or untreated were lysed, and separated on 12.5% SDS-PAGE gels. Then NGAL was probed by antibodies to human NGAL. The bound antibodies were visualised by horseradish peroxidase (HRP)-labelled secondary antibody and 3,3'-diaminobenzidene (DAB). NC, negative control – no first antibody and only HRP-labelled secondary antibody was used.**(c) **NGAL mRNA expression measured by real-time polymerase chain reaction (PCR) analysis. Total RNA was isolated from neutrophils treated with or without GM-CSF for 4 and 18 h. The amount of NGAL mRNA was expressed as a relative value, compared to that of the constitutively expressed housekeeping gene of glyceraldehyde 3-phosphate dehydrogenase (GAPDH). Data are presented as mean ± standard deviation (SD) (n = 3). **(d) **Concentration of NGAL in synovial fluid was measured by ELISA. The horizontal bars indicate the mean values. Each open circle indicates a concentration of NGAL in synovial fluids from individual patients.

The intensity of the NGAL-derived peptide (m/z 1,971.0: monoisotopic ion, and m/z 1,972.0, 1,973.0, 1,974.0: isotopic ions) showed an approximate fourfold increase from the GM-CSF treatment, as shown in Figure 2a. We first investigated whether GM-CSF upregulated the expression of an entire NGAL molecule in neutrophils. Specifically, NGAL in the whole neutrophil lysate was detected by SDS-PAGE followed by western blotting with antibodies to human NGAL. We demonstrated upregulated production of the entire NGAL molecule in neutrophils by GM-CSF (Figure 2b). Further, we next measured the amounts of NGAL mRNA by real-time PCR. As shown in Figure 2c, the level of NGAL mRNA after 4 h of stimulation with GM-CSF increased to be approximately fivefold higher than the level prior to stimulation (p = 0.01). This elevated level almost disappeared after 18 h, which indicated the effect of GM-CSF was transient. Thus, the increased production of the entire NGAL molecule by GM-CSF was demonstrated both at the transcript and protein levels.

### Detection of NGAL in synovial fluid of patients with RA or OA

As stated above, GM-CSF-activated neutrophils increased the production of NGAL *in vitro*. Therefore, we addressed whether the concentration of NGAL in synovial fluid (SF) of patients with RA was elevated by ELISA. We found the concentrations of NGAL in SF were significantly higher in patients with RA than in patients with osteoarthritis (OA) (p = 0.009, Figure 2d), as described previously [27]. Taking this result together with the *in vitro *increase of NGAL in the GM-CSF-stimulated neutrophils, the elevated NGAL levels in the joints of patients with RA would be explained by the activation of neutrophils by GM-CSF.

### Proteome analysis of the effects of NGAL on synoviocytes

**Figure 3:**
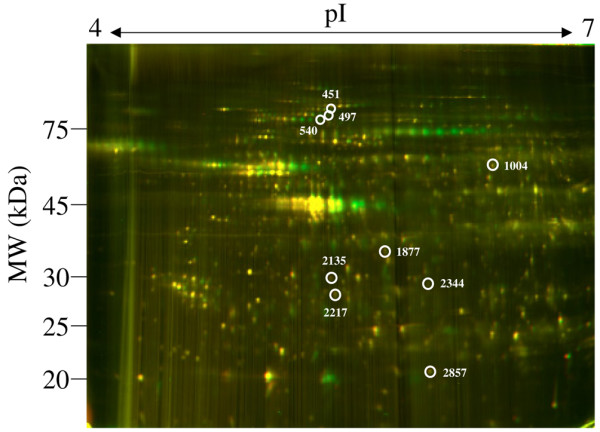
**A representative two-dimensional differential gel electrophoresis (2D-DIGE) analysis of neutrophil gelatinase-associated lipocalin (NGAL)-affected proteins in synoviocytes**. Proteins from synoviocytes treated with NGAL or untreated were labelled separately with Cy5 (green) and Cy3 (red), and then were separated on the same gel using the 2D-DIGE system. Approximately 3,600 protein spots were visualised by laser scanning. On treatment with NGAL, the intensities of 15 and 6 protein spots increased by up to more than 1.5-fold and decreased by less than 1 to 1.5-fold, respectively. Identified protein spots are indicated by open circles. The number near the circle is the spot number, as indicated in Table 4.

Next, we addressed possible roles of the increased NGAL in RA. As reported, NGAL is involved in the allosteric activation of MMP-9 and protection of MMP-9 from degradation [23-25]. However, other functions of NGAL remain to be elucidated. We tried to detect other effects of NGAL on synoviocytes. NGAL did not stimulate synoviocytes to proliferate nor survive (data not shown). Since the concentration of NGAL was found to be high in synovial fluid in RA (Figure 2d), we analysed proteome alteration in synoviocytes from patients with RA by treatment with NGAL *in vitro*. Specifically, proteins extracted from synoviocytes, treated or untreated with NGAL, were separately labelled with two different fluorescent dyes (Cy3 and Cy5) and then analysed by 2D-DIGE, which provided a visual image of proteome differences (Figure 3). For a quantitative assay, equal amounts of proteins from NGAL-treated and untreated synoviocytes were mixed and labelled with Cy3 as a standard. Additionally, each of the two samples was labelled with Cy5 and then was compared with the standard. Approximately 3,600 protein spots were detected on the gel. Out of the detected protein spots, the intensities of 21 protein spots were found to have increased by more than 1.5-fold and 10 decreased by less than 1 to 1.5-fold as a result of the NGAL treatment (Table 3). We tried to identify the 21 increased protein spots, and successfully identified 9 protein spots as shown in Table 4. Among the nine identified proteins, TERA, TG2 and cathepsin D are especially interesting, since they are thought to be involved in promotion of inflammation (as discussed below).

**Table 3:** Number of neutrophil gelatinase-associated lipocalin (NGAL)-affected synoviocyte proteins detected by 2D-DIGE

Change of spot intensities (treated/untreated)	No. of protein spots
3.0 ≤ x	1
2.0 ≤ x < 3.0	3
1.5 ≤ x < 2.0	17
0.67 < x < 1.5	2,245
0.5 < x ≤ 0.67	7
0.33 < x ≤ 0.5	2
x ≤ 0.33	1
Total	2,276

**Table 4:** The identified synoviocyte proteins increased by neutrophil gelatinase-associated lipocalin (NGAL)

Spot no.	Ratio (treated/untreated)	MW (kDa)/pI (observed)	Protein	MW (kDa)/pI (calculated)	Accession no. (Swiss-Prot)
497	4.8	93.3/5.4	Transitional endoplasmic reticulum ATPase	89.0/5.1	[Swiss-Prot:P55072]
451	1.6	95.0/5.5	Actinin 4	104.8/5.3	[Swiss-Prot:O43707]
540	1.6	91.0/5.4	Transglutaminase 2	77.3/5.1	[Swiss-Prot:P21980]
2,217	1.5	29.5/5.6	Cathepsin D	44.5/6.1	[Swiss-Prot:P07339]
1,004	1.6	67.6/6.4	T-complex protein 1 subunit β	57.3/6.0	[Swiss-Prot:P78371]
1,877	1.6	38.9/5.7	Dimethylargininase-2	38.9/5.7	[Swiss-Prot:O95865]
2,135	1.6	32.9/5.4	Prohibitin	29.8/5.6	[Swiss-Prot:P35232]
2,344	1.8	28.0/5.9	Endoplasmic reticulum protein 29	28.0/5.9	[Swiss-Prot:P30040]
2,857	1.5	18.4/6.0	Nucleoside diphosphate kinase A	17.1/5.8	[Swiss-Prot:P15531]

### Confirmation of the upregulation of TERA after the NGAL treatment

**Figure 4:**
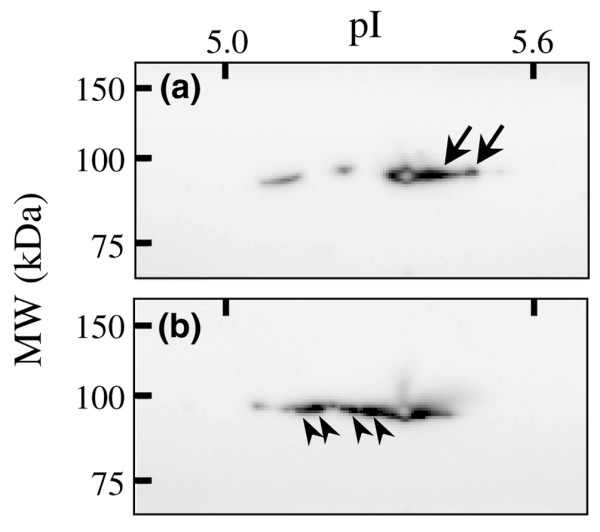
**Western blotting analysis of transitional endoplasmic reticulum ATPase (TERA)**. Synoviocytes prepared from a patient with rheumatoid arthritis (RA) were cultured in the absence **(a) **or the presence **(b) **of neutrophil gelatinase-associated lipocalin (NGAL) for 48 h. Proteins were extracted and separated by two-dimensional electrophoresis (2-DE). The separated proteins were blotted and detected with anti-TERA antibody. Arrows represent the protein spots decreased after the NGAL treatment. Arrowheads represent the protein spots increased after the NGAL treatment.

To confirm the results obtained from the proteomic analysis, we determined whether TERA was increased after NGAL treatment using western blot analysis. This experiment was performed using synoviocytes from a different donor. Proteins were extracted from the synoviocytes treated with or without NGAL, and separated by 2-DE. The separated proteins were blotted onto a membrane and detected with anti-TERA antibody. As shown in Figure 4, a series of protein spots with similar MW but different pI values were detected as TERA. We measured the total amount of chemical luminescence of each sample. The amounts from the NGAL untreated and treated samples were 3.22 × 10^7 ^AU and 6.52 × 10^7 ^AU, respectively. Thus, we have shown NGAL treatment increases the amount of TERA in synoviocytes. Furthermore, 2-DE separation and western blotting revealed that the NGAL treatment decreases protein spots with basic pI (Figure 4a, arrow) and increases protein spots with acidic pI (Figure 4b, arrowhead). These acidic pI shifts of the protein spots without apparent change of MW could be caused by post-translational modifications (PTMs) such as acetylation and/or phosphorylation.

### Effect of NGAL on the proliferation of OUMS-27 and chondrocytes treated with FGF-2 and EGF

**Figure 5:**
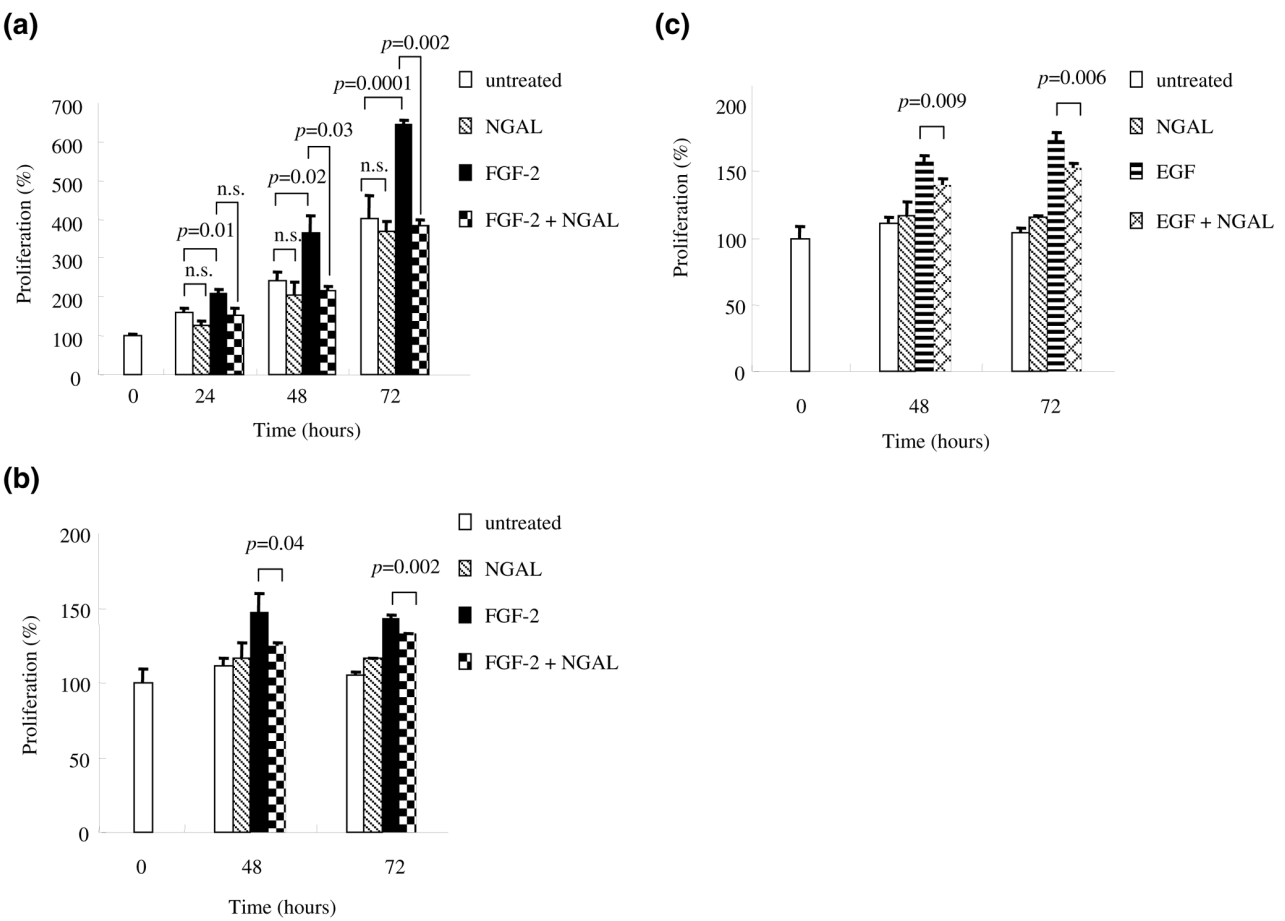
**Effect of neutrophil gelatinase-associated lipocalin (NGAL) on the proliferation of OUMS-27 cells (a) and chondrocytes (b, c)**. A chondrosarcoma cell line (OUMS-27) and chondrocytes from a patient with rheumatoid arthritis (RA) were cultured in medium containing 1 ng/ml fibroblast growth factor (FGF)-2 **(a, b) **or 1 ng/ml epidermal growth factor (EGF) **(c) **and/or 1 μg/ml NGAL. After the time indicated on the x axis, the proliferation of OUMS-27 cells and chondrocytes were measured by dimethylthiazol diphenyltetrazolium bromide (MTT) assay.

Using a chondrosarcoma cell line (OUMS-27) and chondrocytes from a patient with RA, we tried to elucidate the effects of NGAL on proliferation of chondrocytes and on the proliferative action of growth factors. First of all, we tested the action of three growth factors, FGF-2, EGF and TGF-α, on the proliferation of OUMS-27 cells. The proliferation of OUMS-27 cells was significantly upregulated by FGF-2, but not by EGF or TGF-α (data not shown). Therefore, we tested the effects of NGAL with FGF-2. We found, as shown in Figure 5a, NGAL alone did not bring about any significant effects on the proliferation of the cell line. However, the simultaneous addition of NGAL and FGF-2 totally cancelled the proliferative effects of FGF-2 on OUMS-27 cells (Figure 5a). Next, we elucidated the effects of NGAL on the chondrocytes from a patient with RA. Similarly, NGAL alone did not bring about any significant effect on the proliferation of the chondrocytes, but the simultaneous addition of NGAL and FGF-2/EGF cancelled the proliferative effects of FGF-2 (Figure 5b) and of EGF (Figure 5c) on the chondrocytes.

## Discussion

In this study, we investigated effects of GM-CSF on neutrophils by the proteomic approach to understand the role(s) of neutrophils in RA. We have revealed that GM-CSF upregulates the expression of NGAL in neutrophils and that the concentration of NGAL in synovial fluid is elevated significantly in RA patients compared to OA patients. As mentioned earlier, NGAL is reported to be involved in the allosteric activation of MMP-9 and protection of MMP-9 from degradation [23-25], and further, levels of MMP-9 are reported to be high in the serum and synovial fluid of RA patients [26]. Therefore, neutrophils activated by GM-CSF possibly bring about strong activation of MMP-9 by producing NGAL, a pathway that would lead to invasion of immune cells and degradation of cartilage. The activation of MMP-9 is the main known function of NGAL, so we addressed the need to find other effects of NGAL on synoviocytes. By 2D-DIGE proteomic analysis, we identified nine proteins whose expression is upregulated in synoviocytes by NGAL.

Of the nine identified proteins, three (TG2, cathepsin D and TERA) were interesting for the following reasons. First, TG2 belongs to a family of calcium-dependent enzymes which catalyse the acyl transfer reaction between the γ-carboxamide group of a protein-bound glutamine residue and the primary amine group of either a protein-bound lysine residue or other polyamine molecules [28]. Although formation and remodelling of the extracellular matrix [29] are well investigated functions of TG2, intracellular roles have been highlighted only recently. Specifically, TG2 has been reported to activate nuclear factor (NF)κB that contributed to the progression of inflammatory diseases independently of IκB kinase activation by polymerising IκB [30]. Further, TG2 has been reported to serve as an inhibitor of apoptosis of cells by crosslinking of caspase 3 [31]. Taking these reports together with our data, the increase of TG2 by NGAL may contribute to activation of NFκB in synoviocytes and their proliferation in RA.

The second protein of interest was cathepsin D, an aspartic protease. Cathepsin D has been reported to play important roles in the release T cell epitopes from protein antigens for presentation by major histocompatibility complex (MHC) class II molecules [32,33]. Further, synovial cells in patients with RA are known to aberrantly express MHC class II molecules and to act as antigen-presenting cells [34-36]. Thereby the increase of cathepsin D may promote immune reaction in the joints. Cathepsin D is associated with the proliferation, invasion and metastasis of tumour cells. In fact, cathepsin D has been reported to correlate directly with the prognosis of patients with cancer of various organs [37-40]. Additionally, cathepsin D has been reported to be expressed in synovial tissue of patients with RA at a high level compared to that with OA [41]. Thus, the high expression of cathepsin D in RA would be involved in the enhancement of aberrant immunological reactions as well as the enhancement of proliferation or invasion of synoviocytes of RA.

The third identified protein of interest was TERA, also known as valosin-containing protein. TERA plays a key role in the ubiquitin-dependent proteasome degradation pathway [42]. TERA has been reported to work as an antiapoptotic factor and promote metastasis of tumour cells through constant activation of NFκB *in vitro *[43] and has been reported to play an important role in Akt-mediated signalling of cell survival [44]. In fact, high-level expression of TERA in tumours has been reported to be a poor prognostic marker in patients with colorectal carcinomas [45]. It should be mentioned here that TERA was the protein with the most increased level after NGAL-treatment among the nine identified proteins (Table 4), and PTMs of TERA were changed by the treatment (Figure 4). Therefore, increased amounts and changed PTMs of TERA in the synoviocytes treated by NGAL may also contribute to both inflammation of synovium and proliferation of synovial cells.

Taken together, the increased level of NGAL expressed from GM-CSF-stimulated neutrophils in SF upregulates TG2, cathepsin D and TERA. Thereafter, these three enzymes possibly cause a proliferation of synovial cells and infiltration of inflammatory cells into the synovium, leading synovial cells to the RA state.

Finally, we investigated whether NGAL affected the proliferation of chondrocytes and chondrosarcoma cells. Interestingly, NGAL cancelled the proliferative effect of FGF-2 and EGF on chondrocytes and that of FGF-2 on chondrosarcoma cells, but did not suppress the baseline proliferation of either cell type. This indicates that NGAL inhibits the FGF-2 and EGF signalling pathway in an intracellular or extracellular manner. FGF-2 and EGF, as well as TGF-β and other growth factors, are thought to be important for homeostasis of cartilage. For example, FGF-2 has been reported to play crucial roles in enhancing chondrogenic lineage differentiation in human bone marrow-derived mesenchymal cells [46] and in adipose-derived mesenchymal cells [47]. Therefore, the cancellation of the proliferative effects of FGF-2 and EGF by NGAL would contribute to the degradation of cartilage in RA.

## Conclusion

Our study implicates the follwing chain reaction in RA: GM-CSF-stimulated neutrophils increase production of NGAL, then NGAL enhances immunological and/or cell biological activation of synoviocytes through TG2, cathepsin D, and TERA. Further, NGAL abolishes chondrocyte proliferation by FGF-2 and EGF. NGAL may therefore be a crucial pathogenic factor and also a therapeutic target of RA.

## Abbreviations

CHAPS: 3-((3-cholamidopropyl)dimethylammonio)propanesulfonate; DAB: 3,3'-diaminobenzidene; 2D-DIGE: two-dimensional differential gel electrophoresis; 2-DE: two-dimensional electrophoresis; DTT: dethiothreitol; EGF: epidermal growth factor; ERK: extracellular signal-regulated kinase; FBS: foetal bovine serum; FGF: fibroblast growth factor; GAPDH: glyceraldehyde 3-phosphate dehydrogenase; G-CSF: granulocyte colony-stimulating factor; GM-CSF: granulocyte-macrophage colony-stimulating factor; HRP: horseradish peroxidase; IL: interleukin; IEF: isoelectric focusing; MALDI-TOF MS: matrix-assisted laser desorption ionisation-time-of-flight mass spectrometer; MMP: matrix metalloproteinase; NGAL: neutrophil gelatinase-associated lipocalin; NADPH: nicotinamide adenine dinucleotide phosphate; OA: osteoarthritis; PBS: phosphate buffered saline; PCR: polymerase chain reaction; PTM: post-translational modification; RA: rheumatoid arthritis; SF: synovial fluid; TG2: transglutaminase 2; TERA: transitional endoplasmic reticulum ATPase; TFA: trifluoroacetic acid.

## Competing interests

The authors declare that they have no competing interests.

## Authors' contributions

MK carried out over half of the experiments. MA carried out additional experiments. KO participated in general supervision of the experiments by MK and MA. YK, HN and KM prepared clinical samples. YX and SS gave specific aid on the proteome analysis. MSK and NS participated in preparation of the manuscript. KY was an adviser from the standpoint of an orthopaedic rheumatologist. TK was responsible for the planning of the study and directing of the study team.

## References

[B1] McInnes IB, Schett G (2007). Cytokines in the pathogenesis of rheumatoid arthritis. Nat Rev Immunol.

[B2] Gavioli R, Risso A, Smilovich D, Baldissarro I, Capra MC, Bargellesi A, Cosulich ME (1992). CD69 molecule in human neutrophils: its expression and role in signal-transducing mechanisms. Cell Immunol.

[B3] Babior BM (1984). Oxidants from phagocytes: agents of defense and destruction. Blood.

[B4] Smith JA (1994). Neutrophils, host defense, and inflammation: a double-edged sword. J Leukoc Biol.

[B5] Beaulieu AD, McColl SR (1994). Differential expression of two major cytokines produced by neutrophils, interleukin-8 and the interleukin-1 receptor antagonist, in neutrophils isolated from the synovial fluid and peripheral blood of patients with rheumatoid arthritis. Arthritis Rheum.

[B6] Konttinen YT, Lindy O, Kemppinen P, Saari H, Suomalainen K, Vauhkonen M, Lindy S, Sorsa T (1991). Collagenase reserves in polymorphonuclear neutrophil leukocytes from synovial fluid and peripheral blood of patients with rheumatoid arthritis. Matrix.

[B7] Lieschke GJ, Grail D, Hodgson G, Metcalf D, Stanley E, Cheers C, Fowler KJ, Basu S, Zhan YF, Dunn AR (1994). Mice lacking granulocyte colony-stimulating factor have chronic neutropenia, granulocyte and macrophage progenitor cell deficiency, and impaired neutrophil mobilization. Blood.

[B8] Metcalf D, Begley CG, Williamson DJ, Nice EC, De Lamarter J, Mermod JJ, Thatcher D, Schmidt A (1987). Hemopoietic responses in mice injected with purified recombinant murine GM-CSF. Exp Hematol.

[B9] Dang PM, Stensballe A, Boussetta T, Raad H, Dewas C, Kroviarski Y, Hayem G, Jensen ON, Gougerot-Pocidalo MA, El-Benna J (2006). A specific p47phox-serine phosphorylated by convergent MAPKs mediates neutrophil NADPH oxidase priming at inflammatory sites. J Clin Invest.

[B10] Nolan B, Duffy A, Paquin L, De M, Collette H, Graziano CM, Bankey P (1999). Mitogen-activated protein kinases signal inhibition of apoptosis in lipopolysaccharide-stimulated neutrophils. Surgery.

[B11] Wei S, Liu JH, Epling-Burnette PK, Gamero AM, Ussery D, Pearson EW, Elkabani ME, Diaz JI, Djeu JY (1996). Critical role of Lyn kinase in inhibition of neutrophil apoptosis by granulocyte-macrophage colony-stimulating factor. J Immunol.

[B12] Atzeni F, Schena M, Ongari AM, Carrabba M, Bonara P, Minonzio F, Capsoni F (2002). Induction of CD69 activation molecule on human neutrophils by GM-CSF, IFN-γ, and IFN-α. Cell Immunol.

[B13] Xu WD, Firestein GS, Taetle R, Kaushansky K, Zvaifler NJ (1989). Cytokines in chronic inflammatory arthritis. II. Granulocyte-macrophage colony-stimulating factor in rheumatoid synovial effusions. J Clin Invest.

[B14] Bell AL, Magill MK, McKane WR, Kirk F, Irvine AE (1995). Measurement of colony-stimulating factors in synovial fluid: potential clinical value. Rheumatol Int.

[B15] Xiang Y, Matsui T, Matsuo K, Shimada K, Tohma S, Nakamura H, Masuko K, Yudoh K, Nishioka K, Kato T (2007). Comprehensive investigation of disease-specific short peptides in sera from patients with systemic sclerosis: complement C3f-des-arginine, detected predominantly in systemic sclerosis sera, enhances proliferation of vascular endothelial cells. Arthritis Rheum.

[B16] Axelsson L, Bergenfeldt M, Ohlsson K (1995). Studies of the release and turnover of a human neutrophil lipocalin. Scand J Clin Lab Invest.

[B17] Segal AW, Jones OT (1980). Rapid incorporation of the human neutrophil plasma membrane cytochrome *b *into phagocytic vacuoles. Biochem Biophys Res Commun.

[B18] Kunisada T, Miyazaki M, Mihara K, Gao C, Kawai A, Inoue H, Namba M (1998). A new human chondrosarcoma cell line (OUMS-27) that maintains chondrocytic differentiation. Int J Cancer.

[B19] Altman R, Asch E, Bloch D, Bole G, Borenstein D, Brandt K, Christy W, Cooke TD, Greenwald R, Hochberg M (1986). Development of criteria for the classification and reporting of osteoarthritis. Classification of osteoarthritis of the knee. Diagnostic and Therapeutic Criteria Committee of the American Rheumatism Association. Arthritis Rheum.

[B20] Arnett FC, Edworthy SM, Bloch DA, McShane DJ, Fries JF, Cooper NS, Healey LA, Kaplan SR, Liang MH, Luthra HS (1988). The American Rheumatism Association 1987 revised criteria for the classification of rheumatoid arthritis. Arthritis Rheum.

[B21] Matrix Science. http://www.matrixscience.com.

[B22] Shimada S, Nakamura M, Tanaka Y, Tsutsumi K, Katano M, Masuko K, Yudoh K, Koizuka I, Kato T (2007). Crosslinking of the CD69 molecule enhances S100A9 production in activated neutrophils. Microbiol Immunol.

[B23] Tschesche H, Zolzer V, Triebel S, Bartsch S (2001). The human neutrophil lipocalin supports the allosteric activation of matrix metalloproteinases. Eur J Biochem.

[B24] Yan L, Borregaard N, Kjeldsen L, Moses MA (2001). The high molecular weight urinary matrix metalloproteinase (MMP) activity is a complex of gelatinase B/MMP-9 and neutrophil gelatinase-associated lipocalin (NGAL). Modulation of MMP-9 activity by NGAL. J Biol Chem.

[B25] Gupta K, Shukla M, Cowland JB, Malemud CJ, Haqqi TM (2007). Neutrophil gelatinase-associated lipocalin is expressed in osteoarthritis and forms a complex with matrix metalloproteinase 9. Arthritis Rheum.

[B26] Gruber BL, Sorbi D, French DL, Marchese MJ, Nuovo GJ, Kew RR, Arbeit LA (1996). Markedly elevated serum MMP-9 (gelatinase B) levels in rheumatoid arthritis: a potentially useful laboratory marker. Clin Immunol Immunopathol.

[B27] Blaser J, Triebel S, Tschesche H (1995). A sandwich enzyme immunoassay for the determination of neutrophil lipocalin in body fluids. Clin Chim Acta.

[B28] Chen JS, Mehta K (1999). Tissue transglutaminase: an enzyme with a split personality. Int J Biochem Cell Biol.

[B29] Aeschlimann D, Thomazy V (2000). Protein crosslinking in assembly and remodelling of extracellular matrices: the role of transglutaminases. Connect Tissue Res.

[B30] Lee J, Kim YS, Choi DH, Bang MS, Han TR, Joh TH, Kim SY (2004). Transglutaminase 2 induces nuclear factor-κB activation via a novel pathway in BV-2 microglia. J Biol Chem.

[B31] Yamaguchi H, Wang HG (2006). Tissue transglutaminase serves as an inhibitor of apoptosis by cross-linking caspase 3 in thapsigargin-treated cells. Mol Cell Biol.

[B32] Rodriguez GM, Diment S (1992). Role of cathepsin D in antigen presentation of ovalbumin. J Immunol.

[B33] Hewitt EW, Treumann A, Morrice N, Tatnell PJ, Kay J, Watts C (1997). Natural processing sites for human cathepsin E and cathepsin D in tetanus toxin: implications for T cell epitope generation. J Immunol.

[B34] Chin JE, Winterrowd GE, Krzesicki RF, Sanders ME (1990). Role of cytokines in inflammatory synovitis. The coordinate regulation of intercellular adhesion molecule 1 and HLA class I and class II antigens in rheumatoid synovial fibroblasts. Arthritis Rheum.

[B35] Firestein GS, Paine MM, Littman BH (1991). Gene expression (collagenase, tissue inhibitor of metalloproteinases, complement, and HLA-DR) in rheumatoid arthritis and osteoarthritis synovium. Quantitative analysis and effect of intraarticular corticosteroids. Arthritis Rheum.

[B36] Boots AM, Wimmers-Bertens AJ, Rijnders AW (1994). Antigen-presenting capacity of rheumatoid synovial fibroblasts. Immunology.

[B37] Tandon AK, Clark GM, Chamness GC, Chirgwin JM, McGuire WL (1990). Cathepsin D and prognosis in breast cancer. N Engl J Med.

[B38] Otto FJ, Goldmann T, Biess B, Lippold A, Suter L, Westhoff U (1999). Prognostic classification of malignant melanomas by combining clinical, histological, and immunohistochemical parameters. Oncology.

[B39] Allgayer H, Babic R, Grutzner KU, Beyer BC, Tarabichi A, Wilhelm Schildberg F, Heiss MM (1997). An immunohistochemical assessment of cathepsin D in gastric carcinoma: its impact on clinical prognosis. Cancer.

[B40] Arao J, Fukui H, Ono Y, Ueda Y, Chiba T, Fujimori T (2000). Immunohistochemical localization of cathepsin D in colorectal tumors. Dis Colon Rectum.

[B41] Keyszer GM, Heer AH, Kriegsmann J, Geiler T, Trabandt A, Keysser M, Gay RE, Gay S (1995). Comparative analysis of cathepsin L, cathepsin D, and collagenase messenger RNA expression in synovial tissues of patients with rheumatoid arthritis and osteoarthritis, by *in situ *hybridization. Arthritis Rheum.

[B42] Dai RM, Chen E, Longo DL, Gorbea CM, Li CC (1998). Involvement of valosin-containing protein, an ATPase Co-purified with IκBα and 26 S proteasome, in ubiquitin-proteasome-mediated degradation of IκBα. J Biol Chem.

[B43] Asai T, Tomita Y, Nakatsuka S, Hoshida Y, Myoui A, Yoshikawa H, Aozasa K (2002). VCP (p97) regulates NFκB signaling pathway, which is important for metastasis of osteosarcoma cell line. Jpn J Cancer Res.

[B44] Vandermoere F, El Yazidi-Belkoura I, Slomianny C, Demont Y, Bidaux G, Adriaenssens E, Lemoine J, Hondermarck H (2006). The valosin-containing protein (VCP) is a target of Akt signaling required for cell survival. J Biol Chem.

[B45] Yamamoto S, Tomita Y, Hoshida Y, Sakon M, Kameyama M, Imaoka S, Sekimoto M, Nakamori S, Monden M, Aozasa K (2004). Expression of valosin-containing protein in colorectal carcinomas as a predictor for disease recurrence and prognosis. Clin Cancer Res.

[B46] Solchaga LA, Penick K, Porter JD, Goldberg VM, Caplan AI, Welter JF (2005). FGF-2 enhances the mitotic and chondrogenic potentials of human adult bone marrow-derived mesenchymal stem cells. J Cell Physiol.

[B47] Chiou M, Xu Y, Longaker MT (2006). Mitogenic and chondrogenic effects of fibroblast growth factor-2 in adipose-derived mesenchymal cells. Biochem Biophys Res Commun.

